# Toward selective amyloid clearance through rational nanomedicine design

**DOI:** 10.1002/ctm2.70859

**Published:** 2026-10-05

**Authors:** Tianyi Qi, Jie Zhuang, Yuejuan Cao, Xinglu Huang

**Affiliations:** ^1^ Department of Cardiology Tianjin Union Medical Centre The First Affiliated Hospital of Nankai University Tianjin China; ^2^ Key Laboratory of Bioactive Materials for the Ministry of Education, College of Life Sciences Academy for Advanced Interdisciplinary Studies Nankai University Tianjin China

1

With the accelerating global ageing population, Alzheimer's disease (AD) has emerged as a major medical challenge with profound implications for human health and society. Yet, the blood–brain barrier, complex disease pathophysiology, and lack of effective targeted interventions remain major obstacles to the treatment of AD. Accumulation of amyloid‐β (Aβ) remains a defining pathological feature of AD and one of its most intensively pursued therapeutic targets. Recent clinical studies of Aβ‐directed monoclonal antibodies have shown that cerebral amyloid reduction can be accompanied by a measurable slowing of clinical decline.^1^, ^2^ At the same time, the clinical benefits of anti‐Aβ therapies remain incomplete, highlighting the need to better understand how therapeutic agents engage and remodel disease‐relevant Aβ assemblies. Nanomedicine offers opportunities to engineer such interactions at the molecular level, but translating these opportunities into effective therapies requires a clearer link between molecular recognition, structural intervention and therapeutic outcome.^3^, ^4^ Together, these considerations underscore the need for therapeutic strategies that can selectively engage disease‐relevant Aβ assemblies and translate molecular recognition into effective amyloid remodelling in the brain.

To address this need, we recently developed _Aβ3_FTn^Au^, a computationally guided protein‐gold hybrid nanostructure designed to combine selective recognition of aggregated Aβ with structural disruption within a defined molecular framework (Figure 1).^5^ Aβ‐binding peptide sequences were computationally screened and displayed on a human ferritin nanocage to establish multivalent Aβ recognition, while the nanocage‐associated gold component provided an additional structural intervention. This design links target recognition with aggregate disruption rather than treating them as separate functions. Together, these findings illustrate how molecularly guided design may enable therapeutic agents to recognise disease‐relevant Aβ assemblies and directly intervene in their pathological state.

**FIGURE 1:**
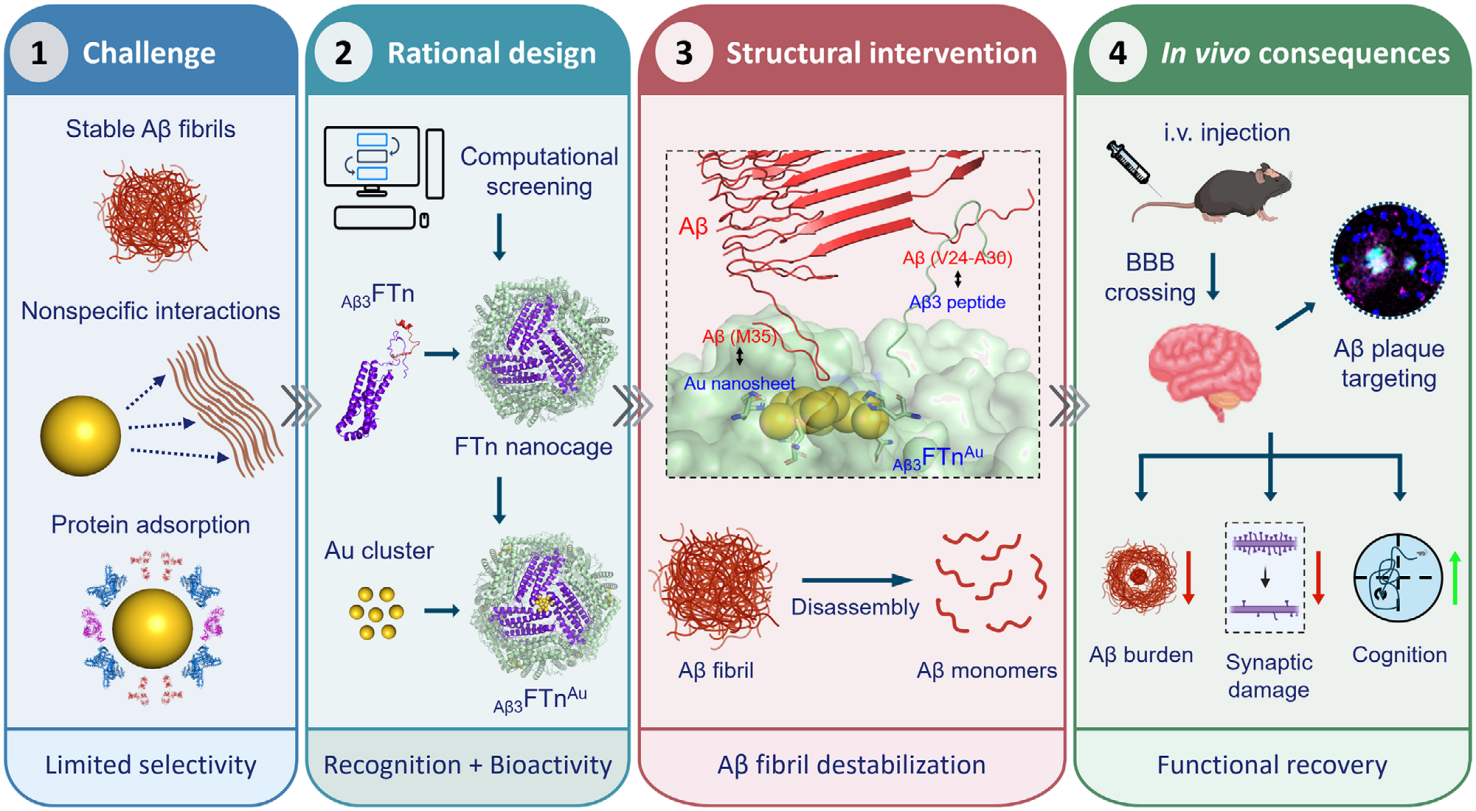
Rational design and therapeutic concept of _Aβ3_FTn^Au^ for targeted amyloid clearance. Conventional nanomaterial‐based approaches can be limited by the structural stability of pre‐existing amyloid‐β (Aβ) fibrils, nonspecific nanomaterial interactions and protein adsorption in biological environments. _Aβ3_FTn^Au^ integrates computationally selected Aβ‐recognition motifs with a ferritin nanocage containing confined gold nanoclusters, coupling selective Aβ recognition with a bioactive structural interface. Interactions involving the Aβ3 peptide and the gold‐coordinating H118/T122/C130 region promote engagement with fibrillar Aβ and destabilisation of the fibril structure, including interactions around Aβ Met35. Following systemic administration, _Aβ3_FTn^Au^ crosses the blood‐brain barrier, targets cerebral Aβ plaques and is associated with reduced amyloid burden, restored synaptic function and improved cognitive performance in 5×FAD mice.

Structural, mutational and computational analyses revealed how the nanostructure engages Aβ through coordinated molecular interactions, with the recognition motif and gold‐associated interface contributing to sustained binding and fibril destabilisation.^5^ Molecular dynamics simulations suggested that multivalent recognition enables sustained engagement with the fibril interface, followed by interfacial interactions that perturb β‐sheet organisation and favour progressive dissociation of peripheral Aβ molecules. Together, these mechanistic observations connect the molecular architecture of _Aβ3_FTn^Au^ with selective Aβ recognition and fibril destabilisation, providing a structural basis for its anti‐amyloid activity. More broadly, these findings suggest that integrating structural biology and molecular modelling into therapeutic design may help identify how pathological protein assemblies can be selectively engaged and remodelled.

A key question was whether this molecularly defined activity could be retained in a disease‐relevant in vivo setting. Following systemic administration in 5×FAD mice, _Aβ3_FTn^Au^ reached the brain and colocalised with cerebral Aβ deposits. Repeated treatment reduced both soluble and insoluble Aβ and plaque burden, accompanied by restoration of synaptic function and improved cognitive performance.^5^ These effects were associated with recovery of long‐term potentiation and dendritic spine density, together with reduced microglial activation and attenuation of lipid‐associated synaptic pruning signatures. Collectively, these findings support the broader concept that interventions against established Aβ pathology should be evaluated not only by their ability to reduce amyloid burden, but also by their capacity to preserve neuronal and synaptic function.

The translational significance of these findings lies in the possibility of directly remodelling established fibrillar Aβ, offering a complementary mode of intervention to antibody‐mediated amyloid removal. Rather than focusing solely on reducing amyloid burden, this strategy highlights the potential value of intervening directly at the level of pathological Aβ assemblies and their structural integrity. The accompanying preservation of synaptic function and cognitive performance in vivo further supports the concept that effective remodelling of established Aβ pathology may be linked to disease‐relevant functional benefits. More broadly, these findings suggest that the molecular architecture of pathological protein assemblies could be considered more explicitly when developing therapeutic strategies for neurodegenerative diseases.

Several challenges will need to be addressed before this strategy can be translated beyond proof‐of‐concept studies. The current work primarily targets highly aggregated fibrillar Aβ, whereas Aβ in human AD exists as a heterogeneous spectrum of soluble and insoluble assemblies with distinct biological properties.^6^ An important clinical question is therefore not simply whether Aβ can be removed, but which Aβ assemblies should be targeted, at what stage of disease, and to what extent such remodelling can translate into durable neuronal benefit. Future studies will need to integrate computational modelling, structural analysis and disease pathology to identify the Aβ species and disease contexts most amenable to targeted intervention. At the same time, longer‐term studies will be required to define safety, pharmacological behaviour, optimal dosing and the durability of therapeutic benefit in clinically relevant models. Ultimately, such efforts may establish a more predictable framework for targeting pathological protein assemblies, in which molecular recognition, structural remodelling and therapeutic outcome are considered as interconnected components of intervention.

## AUTHOR CONTRIBUTIONS

Tianyi Qi, Jie Zhuang, Yuejuan Cao, and Xinglu Huang wrote the paper.

## CONFLICT OF INTEREST STATEMENT

The authors declare no conflict of interest.

## ETHICS STATEMENT

Not applicable. This Invited Letter does not report any new studies involving human participants or animal subjects.
